# Deep-Learning-based Automatic Segmentation and Quantitative Measurement of Normal Spleen in Chinese Adults

**DOI:** 10.2174/0115734056472787260516112449

**Published:** 2026-05-21

**Authors:** Tian Tan, Yaofeng Zhang, Xiaodong Zhang, Xiaoying Wang

**Affiliations:** 1 Department of Radiology, Peking University First Hospital, No.8, Xishiku Street, Xicheng District, Beijing 100034, China; 2 Beijing Smart Tree Medical Technology Co. Ltd., Beijing, China

**Keywords:** Deep learning, Spleen segmentation, Quantitative measurement, Computed tomography (CT), Chinese adults, Artificial Intelligence

## Abstract

**Introduction:**

The spleen is a key immune organ whose volume and attenuation can change in inflammatory, infectious, and hematologic diseases. In routine practice, splenic enlargement is often assessed using semi-quantitative linear criteria, such as rib-unit-based estimates, which can miss modest but clinically relevant volume changes. While deep learning enables reliable organ segmentation on CT, large-scale descriptions of normal splenic morphometrics in Chinese adults, including age- and sex-related patterns, remain limited.

**Materials and Methods:**

This study developed a 3D V-Net for automated spleen segmentation, and trained it on 2,856 CT examinations from four public datasets: Abdomen CT-1K, FLARE23, AMOS, and the RSNA 2023 Abdominal Trauma dataset. The model’s performance was assessed using the Dice similarity coefficient (DSC), volume similarity (VS), Hausdorff distance (HD), and average Hausdorff distance. For morphometric analysis and institutional validation, this model was then applied to 1,520 CT examinations in 2023 of individuals with a clinically and radiologically normal spleen, comprising a total of 3,490 CT images across phases (NOC/AP/PVP/DP), from which spleen volume, mean CT value, and three-dimensional diameters were extracted.

**Results:**

On the public test set, the model achieved a Dice similarity coefficient (DSC) of 0.982 [interquartile range (IQR): 0.975-0.988], a volumetric similarity (VS) of 0.995 [IQR: 0.991-0.998], and a Hausdorff distance (HD) of 3.047 mm [IQR: 2.578-4.653 mm]. Within the institutional cohort, the median spleen volume was significantly greater in males (213.74 cm^3^ [IQR: 158.40-284.88]) than in females (163.70 cm^3^ [IQR: 125.99-217.72]; *p* < 0.001). Age-related volumetric patterns also differed by sex: males exhibited an inverted U-shaped trajectory, peaking at 273.75 ± 92.96 cm^3^ in the 28-37 year age group, while females displayed a bimodal pattern, peaking at 225.01 ± 65.53 cm^3^in the 18-27 year age group. Furthermore, splenic attenuation was significantly higher in females across the arterial, portal venous, and delayed contrast phases (all *p* < 0.001).

**Discussion:**

The 3D V-Net enabled accurate and efficient spleen segmentation on CT, which permitted automated and reproducible measurement of splenic morphometrics. The observed sex- and age-specific patterns in spleen volume and attenuation likely reflect the combined effects of physiology, including hormonal status, as well as metabolic and body-composition factors. Reported differences across populations suggest that splenic reference values may not be directly transferable between ethnic groups, underscoring the need for population-calibrated morphometric data. Limitations of this study include potential residual confounding, incomplete adjustment for anthropometric covariates, and validation within a single institutional center.

**Conclusion:**

This study establishes a practical deep learning-based pipeline for automated spleen segmentation and standardized morphometric extraction on CT in Chinese adults. The resulting population-specific reference distributions may support more objective interpretation of spleen size and attenuation in routine practice and provide a foundation for future disease-oriented applications. Further multicenter validation is warranted.

## 1 INTRODUCTION

The spleen, located in the left upper quadrant, is the largest lymphoid organ and serves central functions in immune surveillance and blood filtration [1]. Splenic volume alterations are associated with inflammatory, infectious, metabolic, and hematologic disorders [2-6], and volumetric change has been investigated as an imaging biomarker for treatment response and prognosis in contexts such as immunotherapy for metastatic malignancy [3], maintenance chemotherapy [4], and transcatheter arterial chemoembolization for hepatocellular carcinoma [5]. Spleen volume also aids risk stratification in conditions including chronic renal failure on hemodialysis [6], advanced liver fibrosis [7], and esophageal variceal hemorrhage [8]. These associations underscore the need for accurate and reproducible spleen volumetry in clinical decision-making.

CT depicts both diffuse and focal abnormalities in splenic attenuation, and CT-derived volumetry correlates well with reference measurements [9, 10]. In routine practice, however, spleen size is seldom quantified; splenomegaly is typically assessed using simplified linear surrogates, such as a transverse splenic diameter exceeding five rib units on axial images. Such semi-quantitative criteria may miss early or subtle volume changes and can reduce the consistency of clinical assessment.

The rapid development of artificial intelligence in medical imaging has enabled deep learning-based segmentation of many abdominal organs, permitting automated extraction of quantitative features like organ volume, dimensions, and attenuation [11-15]. For the spleen, an accurate segmentation model can deliver fast, objective morphometric measurements-including volume, mean CT value, and diameters-that complement conventional qualitative interpretation. Robust spleen segmentation also forms a technical foundation for downstream tasks, such as automated splenomegaly screening and focal lesion analysis.

Previous studies have described age-related trends in spleen volume [16-18], but many relied on manual or semi-automated measurements and did not employ modern deep learning segmentation in large cohorts of healthy individuals. Given the growing use of spleen volume as a quantitative imaging biomarker, automated population-specific reference data are required. Here, we trained a 3D V-Net model for automated spleen segmentation on abdominal CT and characterized normal splenic morphometrics across age and sex strata in a Chinese adult cohort. Quantitative imaging frameworks and contemporary deep learning segmentation literature provide the methodological foundation for this work [19-23].

## 2 MATERIALS AND METHODS

### 2.1 Patients

This retrospective study received approval from the Institu-tional Review Board of Peking University First Hospital (IRB No. 2024 (222-004)), with a waiver of informed consent.

### 2.2 Training Dataset for a 3D V-Net Segmentation Model

A total of 2,856 CT cases were collected from publicly available datasets-AbdomenCT-1K, FLARE23, AMOS, and RSNA 2023 Abdominal Trauma Detection-for model development. These cases were randomly divided into training (n = 2,292), validation (n = 280), and test (n = 284) sets at an 8:1:1 ratio. This dataset was not employed for quantitative spleen volume analysis. The distribution of CT phases and reconstructed slice thicknesses across data sources is summarized in Table **S1**, and CT acquisition parameter summaries for the development splits are provided in Table **S2**.

### 2.3 Validation Dataset for Segmentation Model and Morphometric Analysis of Spleen

We identified patients who underwent abdominal CT examinations, both non-contrast (NCCT) and contrast-enhanced (CECT), at Peking University First Hospital between January and December 2023. Imaging was performed using one of the seven scanners: iCT 256, SOMATOM Drive, Optima CT680 Expert, SOMATOM Force, NeuViz Prime, Revolution CT, or Discovery CT750 HD, with a reconstructed slice thickness of 1.25 mm or 1 mm. A spleen was classified as normal based on a comprehensive review of clinical and imaging data. Inclusion criteria required patients to be at least 18 years old, with images encompassing the entire spleen and a medical imaging report indicating a normal spleen. Eligible patients also had no clinical history of spleen-related diseases, including hematological disorders (*e.g*., hypersplenism, anemias, leukemia, lymphoma, macroglobulinemia, myelofibrosis), hepatic disorders (*e.g*., cirrhosis, portal hypertension, hepatitis, space-occupying lesions), neoplastic conditions (*e.g*., liver or pancreatic cancer, maintenance chemotherapy for malignancies), or prior splenic surgery. Exclusion criteria comprised image quality defects such as incomplete acquisition, an insufficient scanning range, or significant respiratory motion artifacts; anatomical abnormalities like postsplenectomy absence or major deformity; and imaging abnormalities where re-evaluation suggested abnormal splenic structure, attenuation/enhancement, contour, or size, or suspected focal or diffuse pathology beyond normal anatomic variants (including splenomegaly, focal lesions, infarction, cysts, calcifications, traumatic change, or diffuse heterogeneity). Key acquisition parameters are detailed in Table **S2**. As summarized in Fig. (**1**), 4,277 CT images acquired from January to December 2023 were screened; 787 were excluded (incomplete CT series, inaccurate phases, abnormal spleen, or post-surgical status). The final Dataset 2 included 1,520 CT examinations, comprising a total of 3,490 CT images across phases (NOC n=862, AP n=744, PVP n=947, DP n=937) for morphometric analysis and institutional validation.

### 2.4 Model Training

**Fig. (2):**
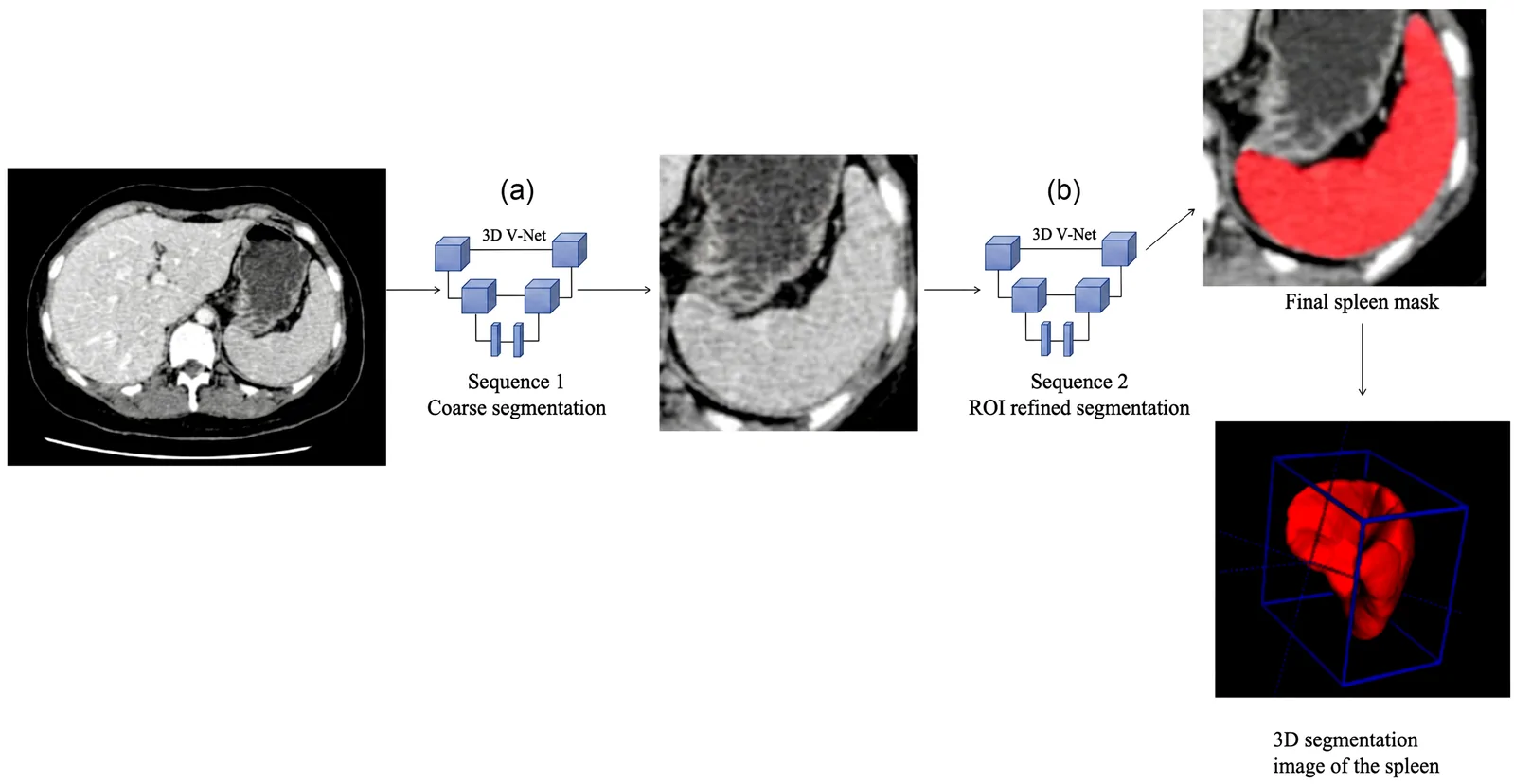
Sequenced segmentation model of the spleen. (**a**) Sequence 1 generates a coarse mask, which is dilated to define the ROI for cropping/resampling. (**b**) Sequence 2 performs ROI-refined segmentation for final mask refinement.

Two sequential spleen segmentation models were implemented using a 3D V-Net architecture. Training was conducted on an NVIDIA Tesla P100-PCIE-16GB GPU with a software environment comprising Python 3.9.1, PyTorch 1.7.1+cu110, OpenCV 4.5.1, NumPy 1.20.0, and SimpleITK 2.0.2. For the sequential-1 model, the public training data were randomly divided into training (n = 2292), validation (n = 280), and test (n = 284) sets in an 8:1:1 ratio. Preprocessing involved windowing (width = 30 HU, level = 300 HU), removal of invalid regions, and resampling to a size of (128, 192, 224). Data augmentation included smoothing, rotation (-10 to 10 degrees), random noise injection (strength: 0.00001), perspective transformation (0 to 5 degrees), shearing (0 to 5 degrees), image translation (-0.1 to 0.1 in x and y), and 3D random rotation (-10 to 10 degrees per axis). The model was trained with an input size of (128, 192, 224), a batch size of 2, for 400 epochs, and a learning rate of 0.0001. This 3D V-Net used n_filters = 16 and was optimized with the Adam algorithm. For the sequential-2 model, the same 8:1:1 data split was applied. Its preprocessing consisted of windowing (30 HU width, 300 HU level), in-plane label dilation by 5 pixels, cropping to the mask boundary, and resampling to (128, 128, 160). The data augmentation strategy matched that of the first model. Training parameters included an input size of (128, 128, 160), a batch size of 1, 400 epochs, and a learning rate of 0.0001. This network used n_filters = 32 and was also optimized with Adam. Figure **2** illustrates the segmentation process of the 3D V-Net architecture. The models produce spleen masks, enabling the calculation of spleen volume, average CT values, and three-dimensional diameters.

### 2.5 Evaluation of the Segmentation Model

Objective and subjective evaluations were conducted to assess the performance of the spleen segmentation model. The objective evaluation on the model development splits employed the Dice similarity coefficient (DSC), volume similarity (VS), Hausdorff distance (HD), and average HD. Two radiologists also performed a subjective assessment of the segmentation predictions on the institutional data. This assessment evaluated the inclusion of all spleen structures. Segmentation was deemed unsatisfactory if extra-splenic elements-such as the splenic artery, vein, diaphragm, gastric wall, or intestinal tract-were included, or if the missing portion of the spleen label exceeded 5% of its total volume. Segmentation was also considered insufficient if the spleen label extended beyond the organ's boundary by 5% or more of its volume. Images with poor segmentations were manually corrected to produce the final output.

### 2.6 Quantitative Measurements

In the institutional cohort (Dataset 2), spleen segmentation on CT examinations was performed automatically using the model. A junior and a senior radiologist jointly reviewed these automated spleen labels, manually correcting any unsatisfactory segmentations. Manual adjustments ensured that each labeled region of interest (ROI) contained only splenic tissue (Fig. **2a, b**), excluding adjacent structures such as the gastric wall, kidney, or liver. Labels deviating by more than 5% from the actual spleen volume were also corrected. For each ROI, the volume, mean CT value, and three-dimensional diameters were measured. Manual refinement was necessary for 58 of the 3,490 CT images (1.7%), while the remaining 3,432 CT images (98.3%) were accepted without modification.

### 2.7 Statistical Analysis

Statistical analyses were conducted using R version 4.4.2, with a two-sided *P* < 0.05 threshold for significance. The Shapiro-Wilk test assessed data normality. Two-group comparisons employed the independent-samples t-test for normally distributed data and the Mann-Whitney U test otherwise. For multi-group comparisons, one-way ANOVA was applied when assumptions of normality and homogeneity of variance were met; if not, the Kruskal-Wallis test was used. In analyses involving multiple subgroup comparisons, such as those stratified by age and phase, *P*-values were adjusted *via* the Benjamini-Hochberg false discovery rate (FDR) procedure; both raw and FDR-adjusted *P*-values are reported in Tables **S4-S6**. The large magnitude of certain test statistics reflects the scale of rank-based measures (Mann-Whitney U/W) in our substantial sample.

## 3 RESULTS

### 3.1 Spleen Segmentation Model Evaluation

The quantitative performance of the model is summarized in Table **1**. For dataset 1, the model achieved a Dice Similarity Coefficient (DSC) of 0.982 [0.975-0.988], a Volume Similarity (VS) of 0.995 [0.991, 0.998], a Hausdorff Distance (HD) of 3.047 [2.578, 4.653] mm, and an average HD of 0.014 [0.009, 0.019] mm on the test set (N = 284). In dataset 2, two radiologists evaluated the algorithm's segmentation predictions after excluding images with focal or diffuse splenic lesions. The automated masks were accepted without modification in 3,432 of 3,490 CT images (98.3%). Manual refinement was necessary for 58 CT images (1.7%), primarily to address minor boundary omissions or slight spillover into adjacent tissues. Source-stratified segmentation metrics for the aggregated public datasets are detailed in Table **S3**. Post-hoc comparisons across the three development splits are presented in Table **S4**. For the institutional cohort, Table **1** reports metrics using the minimum-maximum range to transparently reflect rare lower-performing outliers, as many cases round to DSC=1.000 when reported to three decimal places.

### 3.2 Morphometric Analysis of Spleen

Quantitative spleen morphometrics across CT phases are summarized in Table **2**. As shown in Table **3**, the spleen volume distribution for males (N = 506) was 213.74 [158.40, 284.88] cm^3^, compared to 163.70 [125.99, 217.72] cm^3^ for females. Overall, spleen volume was significantly higher in males (*P* < 0.001). In the age-stratified analysis, the male-female difference was not significant in the 18-27-year group (t = 1.38, *P* = 0.19). Although the mean values differ, this comparison is underpowered because of the small subgroup sample size (male n=9; female n=20), and the observed difference should be interpreted cautiously. Representative segmentation outputs and the bounding-box–based diameter extraction are illustrated in Fig. (**3a**-**d**). Whereas significant differences were observed in most other age strata (*P* < 0.001 for many comparisons). Male spleen volume followed an inverted U-shaped trajectory, peaking at 28-37 years (273.75 ± 92.96 cm^3^). Female spleen volume exhibited a bimodal pattern, with the highest mean volume occurring at 18-27 years (225.01 ± 65.53 cm^3^) Fig. (**4b**). With advancing age, splenic attenuation showed an initial decline followed by a subsequent increase Fig. (**4c**). The three-dimensional spleen dimensions (x × y × z) for all included patients were 9.2 [8.4, 9.95] cm × 9.5 ± 1.97 cm × 9.4 ± 1.91 cm in males and 8.45 ± 1.09 cm × 8.92 ± 1.83 cm × 8.64 ± 1.67 cm in females Fig. (**4d-f**). Phase-specific attenuation changes are shown in Fig. (**4a**), and age-stratified trends in spleen volume and portal venous phase attenuation are shown in Fig. (**4b** and **c**). Raw and FDR-adjusted *P*-values for the age-stratified sex comparisons are provided in Tables **S5** and **S6**.

## 4 DISCUSSION

Quantitative imaging converts routine medical images into reproducible numerical descriptors to support diagnosis, staging, and longitudinal monitoring. Encoder-decoder convolutional architectures, including U-Net-family networks, now enable practical, automated organ segmentation and the extraction of volumetric and morphometric biomarkers. In this study, our two-stage 3D V-Net provided accurate spleen segmentation, allowing the automated measurement of spleen volume, attenuation (HU), and three-dimensional diameters in a large cohort of Chinese adults with clinically and radiologically normal spleens [19, 21, 22].

An automated spleen morphometry pipeline offers several pragmatic clinical advantages. It reduces the workload of manual slice-by-slice delineation while improving repeatability across readers and over time. Standardized quantitative outputs can complement routine qualitative interpretation, particularly where spleen size and attenuation are clinically relevant, such as in portal hypertension evaluation or oncologic and inflammatory disease monitoring. Furthermore, when integrated with downstream quantitative models, automated morphometrics may support more consistent and individualized interpretation than semi-quantitative linear surrogates alone.

Most published spleen volume reference data originate from non-Chinese populations, and inter-population variability has been documented. Such differences likely relate to baseline anthropometrics, sampling frame, and imaging protocols, arguing against applying a single numeric threshold across settings. Our population-specific morphometric distributions therefore offer a pragmatic reference for Chinese adults, while underscoring that reference values should be calibrated to the target population and acquisition context.

We observed sex- and age-related patterns in spleen volume and attenuation. These trajectories should be interpreted cautiously, as our retrospective dataset lacked complete anthropometric covariates-such as height, weight, BMI, and body surface area-and did not directly measure biological factors like hormonal status or detailed body-composition metrics that may underlie these patterns. Future studies with a standardized collection of anthropometrics and relevant clinical covariates will be needed to disentangle physiologic variation from residual confounding and to derive adjusted reference intervals.

A notable finding was the lower segmentation performance on real-world institutional CT scans compared with the public test set. This gap aligns with domain shift across datasets, likely reflecting scanner and protocol variability, reconstruction differences, and heterogeneous boundary contrast in routine clinical images. To ensure clinical usability, all institutional masks were reviewed by radiologists; only 1.7% (58/3,490) required minor manual refinement, mainly for small boundary omissions or limited spillover into adjacent structures. This quality-control step was implemented to guarantee reliable morphometric measurements in practice and to clarify the extent of human involvement still required.

Finally, to improve technical and clinical generalizability, future work should incorporate multicenter external validation and benchmarking against strong contemporary baselines, such as nnU-Net [24] and other contemporary segmentation frameworks [21, 22]. Such comparisons would clarify performance under heterogeneous acquisition conditions and help define the optimal deployment strategy for spleen morphometry in routine workflows.

## 5 STUDY LIMITATIONS

Several limitations should be acknowledged. First, while individuals with overt conditions known to affect splenic size were excluded, residual confounding from factors such as subclinical infection or metabolic abnormalities may persist and influence the measured volumes. Second, complete structured data for anthropometric covariates, including height, weight, BMI, and BSA, were unavailable in our retrospective institutional dataset, precluding their incorporation into the present analysis; future multicenter studies with standardized anthropometric collection are needed to establish adjusted reference intervals. Third, the single-center origin of our institutional validation cohort and its uneven age distribution across strata may limit the generalizability of the findings. Finally, the model was developed for morphologically normal spleens and may be less robust when applied to anatomic variants or complex disease states; further data from diverse clinical scenarios are required before broader deployment.

## CONCLUSION

In conclusion, the 3D V-Net-based pipeline robustly segmented the spleen and enabled efficient, standardized volumetric assessment from CT scans. The automated morphometric outputs can support more objective evaluation in clinical practice and facilitate large-scale quantitative studies.

## Figures and Tables

**Fig. (1):**
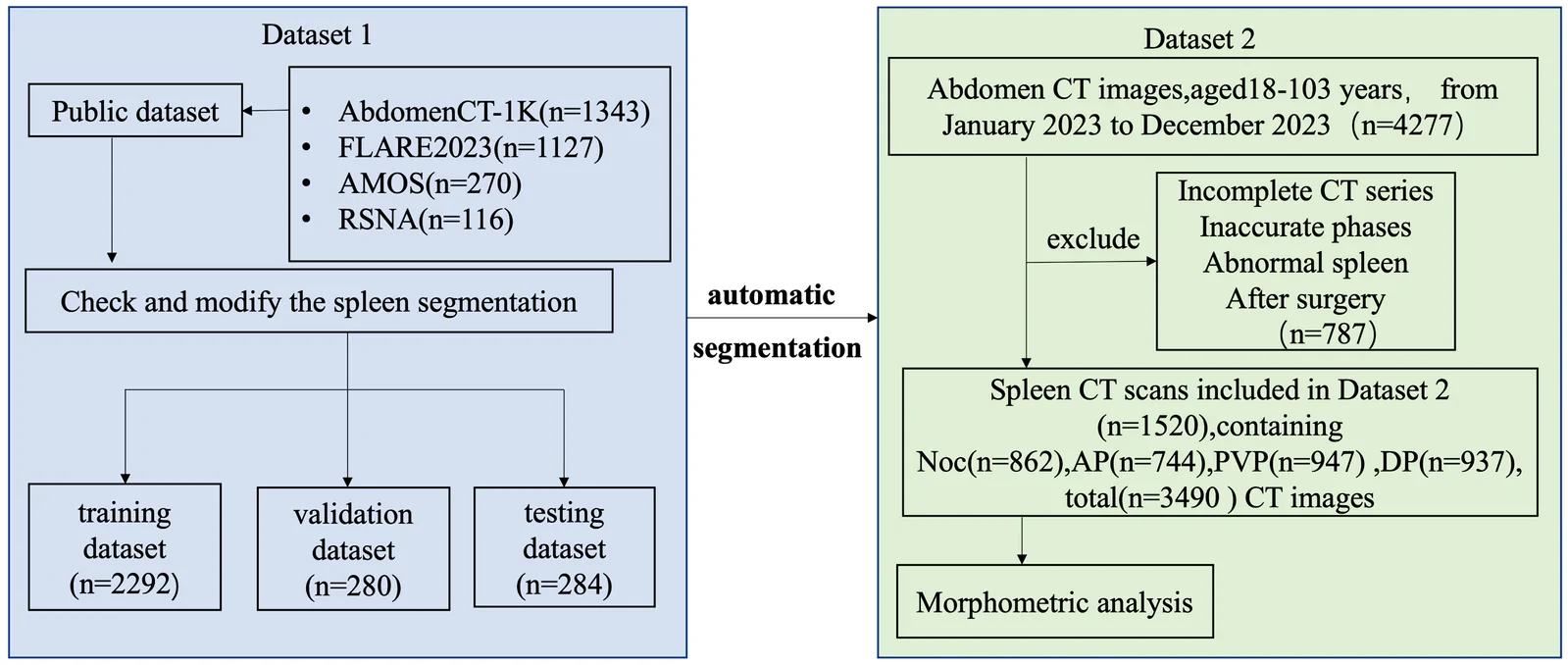
This figure illustrates the image data enrollment and usage. In Dataset 2, 4,277 CT images were initially screened; after exclusions (n=787), 1,520 CT examinations were included, comprising a total of 3,490 CT images across phases (NOC/AP/PVP/DP).

**Fig. (3):**
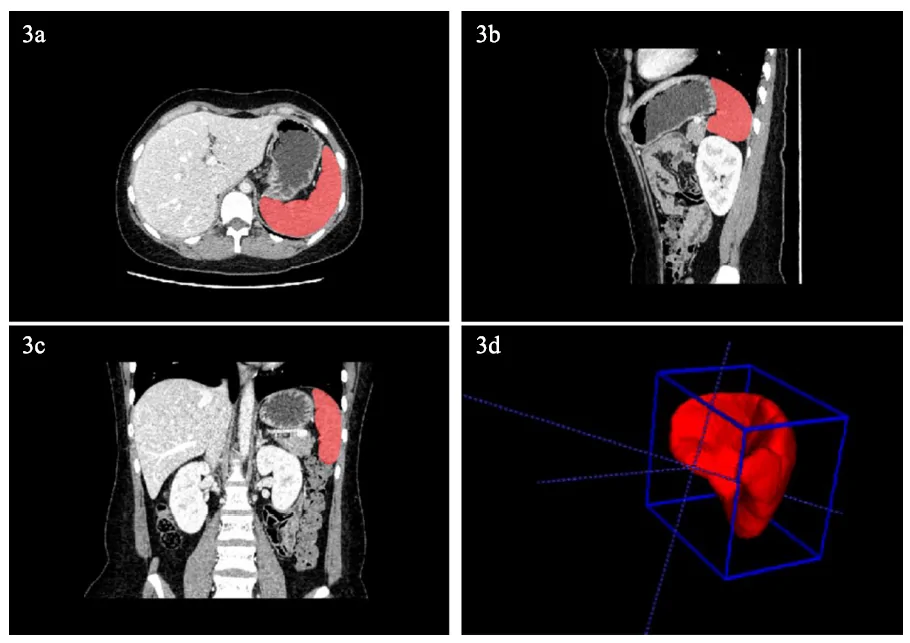
Shows the spleen labeled on a venous phase CT image (**a**) and a schematic illustration of satisfactory segmentation results. The schematic depictions in (**a**-**c**) represent the spleen labels in the axial, coronal, and sagittal planes, respectively. (**d**) demonstrates the application of the minimum volume bounding box algorithm, in which the rectangle's length, width, and height correspond to the three-dimensional dimensions of the spleen.

**Fig. (4):**
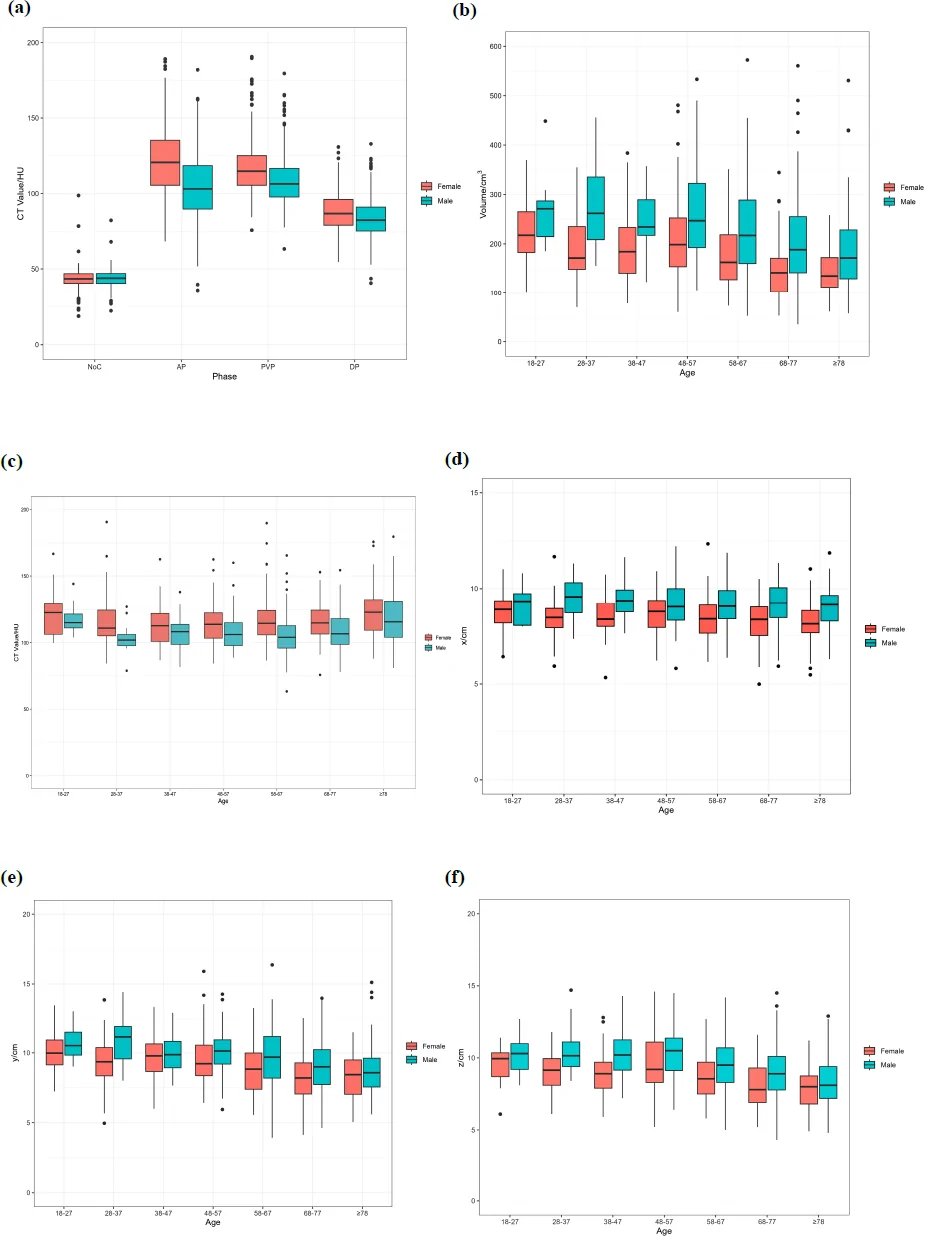
Illustrates the changes in CT value across different phases (**a**). The spleen volume (**b**) and portal venous phase CT value (**c**) are shown across age groups, and the three orthogonal diameters are shown in (**d**-**f**).

**Table 1:** Objective evaluation results of the spleen segmentation model.

Parameter	Institutional Cohort (Dataset 2; n=3,490 CT images)	Testing Set(n=284)	Training Set(n=2292)	Validation Set (n=280)	Statistical Value	*P*-value
DSC	0.736-1	0.982[0.975,0.988]	0.983[0.977,0.987]	0.982[0.974,0.987]	5393.048	<0.001
VS	0.738-1	0.995[0.991,0.998]	0.996[0.992,0.998]	0.996[0.991,0.998]	5313.516	<0.001
HD/mm	0-57.237	3.047[2.578,4.653]	2.839[2.389,3.658]	3.094[2.528,4.713]	5263.445	<0.001
Average HD/mm	0-21.963	0.014[0.009,0.019]	0.013[0.009,0.017]	0.014[0.01,0.021]	5317.603	<0.001

**Note:** Data are described as median [interquartile range] or minimum–maximum. DSC, Dice similarity coefficient; VS, volume similarity; HD, Hausdorff distance.

**Table 2:** Quantitative parameters of the spleen in different phases.

Parameter	All				Noc				AP				PVP				DP			
	Male	Female	Statistical Value	*P*-value	Male	Female	Statistical Value	*P*-value	Male	Female	Statistical Value	*P*-value	Male	Female	Statistical Value	*P*-value	Male	Female	Statistical Value	*P*-value
Count	N=1939	N=1551	-	-	N=473	N=389	-	-	N=427	N=317	-	-	N=506	N=441	-	-	N=533	N=404	-	-
Average CT value/HU	88.52 [61.56,105.09]	96.91 [59.04,116.57]	1297301	<0.001	43.86 [40.37,46.93]	43.43 [40.38,46.8]	96145	0.25	104.17±21.42	120.72 [105.47,135.33]	39127	<0.001	106.34 [97.71,116.64]	114.82 [105.47,125.11]	76560	<0.001	82.35 [75.18,91.01]	86.73 [78.99,96.22]	84697	<0.001
Volume/cm^3^	210.42 [160.55,276.41]	161.68 [125.3,216.02]	2018213	<0.001	206.32 [157.23,274.71]	157.55 [122.56,209.96]	123943	<0.001	204.18 [157.5,270.18]	158.87 [124.47,212.02]	91044	<0.001	213.74 [158.4,284.88]	163.7 [125.99,217.72]	149411.5	<0.001	215.14 [169.59,278.78]	167.94 [127.98,226.22]	143889	<0.001
Diameters/cm	-	-	-	-	-	-	-	-	-	-	-	-	-	-	-	-	-	-	-	-
x	9.18 [8.40,9.95]	8.54 [7.82,9.22]	1999402	<0.001	9.15±1.18	8.48±1.08	8.74	<0.001	9.08±1.18	8.59 [7.8,9.23]	86483.5	<0.001	9.2 [8.4,9.95]	8.45±1.09	151433	<0.001	9.27±1.12	8.65 [7.93,9.28]	143818.5	<0.001
y	9.37 [8.07,10.73]	8.82 [7.58,10.12]	1777732	<0.001	9.31±1.99	8.75±1.89	4.23	<0.001	9.34 [8.21,10.67]	8.78±1.81	82694	<0.001	9.5±1.97	8.92±1.83	4.65	<0.001	9.51±1.9	8.86±1.89	5.13	<0.001
z	9.40 [8.05,10.70]	8.50 [7.40,9.70]	1895192.5	<0.001	9.31±1.92	8.40 [7.40,9.30]	119674	<0.001	9.44±1.95	8.50 [7.50,9.50]	85111.5	<0.001	9.40±1.91	8.64±1.67	6.55	<0.001	9.50 [8.10,10.80]	8.57 [7.60,9.80]	134317.5	<0.001

**Note:** Data conforming to a normal distribution are expressed as mean ± standard deviation, otherwise as median [interquartile range].

**Table 3:** Quantitative parameters of the spleen in different age groups.

		All				18-27				28-37				38-47			
		Male	Female	Statistical Value	*P*-value	Male	Female	Statistical Value	*P*-value	Male	Female	Statistical Value	*P*-value	Male	Female	Statistical Value	*P*-value
Count		N=506	N=441			N=9	N=20			N=16	N=34			N=27	N=38		
Average CT value/HU		106.34[97.71,116.64]	114.82[105.47,125.11]	76560	<0.001	118.16±12.8	122.08±17.34	-0.68	0.5	103.38±10.92	110.87[105.07,124.5]	142	0.01	106.22±14.01	113.12±16.2	-1.83	0.07
Volume/cm^3^		213.74[158.40,284.88]	163.70[125.99,217.72]	149411.5	<0.001	267.56±81.17	225.01±65.53	1.38	0.19	273.75±92.96	183.33±63.42	3.52	<0.001	248.97±62.16	184.15[140.2,233.26]	762	<0.001
Diameters/cm		-	-	-	-	-	-	-	-					-	-	-	-
x		9.20[8.04,9.95]	8.45±1.09	151433	<0.001	9.12±1.04	8.76±1.26	0.8	0.44	9.52±1.1	8.55±1.11	2.89	0.01	9.44±1.05	8.49±1.01	3.65	<0.001
y		9.5±1.97	8.92±1.83	4.65	<0.001	10.71±1.29	10.16±1.62	0.99	0.34	10.89±1.8	9.3±1.81	2.92	0.01	9.87±1.41	9.60±1.69	0.7	0.49
z		9.40±1.91	8.64±1.67	6.55	<0.001	10.28±1.65	9.53±1.34	1.2	0.25	10.46±1.77	8.94±1.41	3	0.01	10.30±1.57	8.95±1.59	3.41	<0.001

**Table:** 

48-57				58-67				68-77				≥78			
Male	Female	Statistical Value	*P*-value	Male	Female	Statistical Value	*P*-value	Male	Female	Statistical Value	*P*-value	Male	Female	Statistical Value	*P*-value
N=74	N=77			N=165	N=116			N=156	N=107			N=59	N=49		
106.05[97.68,114.95]	113.35±14.72	2086	<0.001	103.96[95.88,112.71]	114.51[105.79,124.28]	5568	<0.001	107.97±14.06	115.48±12.62	-4.52	<0.001	117.41±21.06	123.29±19.72	-1.5	0.14
247[192.50,322.56]	198.48[153.55,252.44]	3848	<0.001	217.04[159.9,288.69]	162.5 [127.18, 218.46]	12938	<0.001	188.18[141.21,255.14]	141.23[102.34,170.86]	11992	<0.001	171.27[129.04,228.33]	143.15±50.09	1944	<0.001
9.07[8.34,10.00]	8.72±1.04	3471	0.02	9.13±1.15	8.44±1.06	5.22	<0.001	9.18±1.07	8.27±1.08	6.77	<0.001	8.98±1.08	8.2±1.15	3.63	<0.001
10.04±1.52	9.23[8.36,10.56]	3390	0.04	9.63±2.13	8.78±1.75	3.68	<0.001	9.07±1.89	8.28±1.63	3.58	<0.001	8.57[7.56,9.62]	8.31±1.72	1585.5	0.39
10.39±1.69	9.43±1.84	3.33	<0.001	9.45±1.82	8.70±1.51	3.76	<0.001	8.95±1.89	8.04±1.53	4.28	<0.001	8.42±1.83	7.76±1.56	2.03	0.05

**Note:** Data conforming to a normal distribution are expressed as mean ± standard deviation, otherwise as median [interquartile range].

## Data Availability

The training dataset (Dataset 1) used in this study is publicly available from the following repositories: AbdomenCT-1K (Fully-supervised): https://abdomenct-1k- fully-supervised-learning.grand-challenge.org/ AbdomenCT-1K (Semi-supervised): https://abdomenct-1k- semi-supervised-learning.grand-challenge.org/ AbdomenCT-1K labeled subset (Zenodo): https://zenodo. org/record/5903099 FLARE23: https://codalab.lisn.upsaclay.fr/competitions/ 12239#learn_the_details-dataset AMOS: https://zenodo.org/record/7262581 RSNA 2023 Abdominal Trauma Detection: https://www.kaggle.com/competitions/rsna-2023-abdominal-trauma-detection/data The external validation dataset (Dataset 2), consisting of clinical CT images from the participating institution, is not publicly available due to patient privacy restrictions under the Institutional Review Board (IRB) approval No. 2024 (222-004). However, de-identified data and materials are available from the corresponding author [X.W] upon reasonable request, subject to IRB approval and data usage agreements.

## LIST OF ABBREVIATIONS

NCCT: Non-contrast computed tomography
CECT: Contrast enhanced computed tomography
DSC: Dice similarity coefficient
VS: Volume similarity
HD: Hausdorff distance

## STANDARDS OF REPORTING

STROBE guidelines were followed.
