# Supplementary material for: Deep-Learning-based Automatic Segmentation and Quantitative Measurement of Normal Spleen in Chinese Adults
Source: Curr Med Imaging. 2026 May 21;22:E15734056472787. doi: 10.2174/0115734056472787260516112449 (PMC13602113; doi:10.2174/0115734056472787260516112449)
Supplement: Supplementary file 1 [file CMIM-22-E15734056472787_SD1.pdf]

1875-6603/26 Send Orders for Reprints to reprints@benthamscience.net

BENTHAM SCIENCE
CrossMark

Current Medical Imaging

Content list available at: https://benthamscience.com/journals/cmir

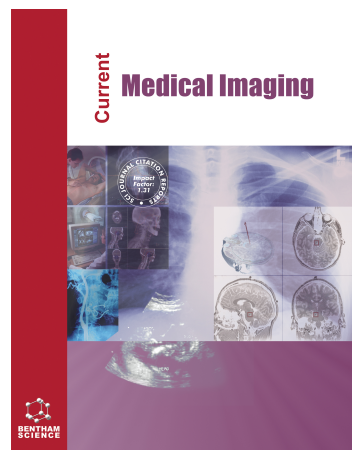

Supplementary Material

Deep-Learning-based Automatic Segmentation and Quantitative Measurement of Normal Spleen in Chinese Adults

Tian Tan[1], Yaofeng Zhang[2], Xiaodong Zhang[1] and Xiaoying Wang[1,*]

[1]*Department of Radiology, Peking University First Hospital, No.8, Xishiku Street, Xicheng District, Beijing 100034, China*
[2]*Beijing Smart Tree Medical Technology Co. Ltd., Beijing, China*

Table S1. Distribution of CT phase and slice thickness in the total cohort and source-specific subsets.

Variables	Total (n = 2856)	Abdomen_CT_date_has_label (n = 944)	Abdomen_CT-1k_Fullysupervised_subtask1 (n = 321)	Abdomen_CT-1k_Semi (n = 38)	AMOS (n = 270)	Flare23 (n = 1121)	RSNA (n = 115)
Phase, n (%)	-	-	-	-	-	-	-
AP	860 (30.11)	285 (30.19)	87 (27.19)	4 (10.53)	73 (27.04)	371 (33.10)	40 (34.78)
DP	379 (13.27)	148 (15.68)	54 (16.88)	16 (42.11)	21 (7.78)	127 (11.33)	13 (11.30)
NOC	146 (5.11)	7 (0.74)	0 (0.00)	3 (7.89)	128 (47.41)	7 (0.62)	1 (0.87)
PVP	1424 (49.86)	504 (53.39)	180 (55.94)	15 (39.47)	48 (17.78)	616 (54.95)	61 (53.04)
Slice thickness (mm), n (%)	-	-	-	-	-	-	-
<1.00	250 (8.75)	106 (11.23)	2 (0.62)	0 (0.00)	0 (0.00)	114 (10.17)	28 (24.35)
>5.00	14 (0.49)	5 (0.53)	1 (0.31)	3 (7.89)	0 (0.00)	5 (0.45)	0 (0.00)
1.00	387 (13.55)	132 (13.98)	72 (22.50)	0 (0.00)	0 (0.00)	180 (16.06)	3 (2.61)
1.25	52 (1.82)	10 (1.06)	3 (0.94)	0 (0.00)	5 (1.85)	22 (1.96)	12 (10.43)
1.50	78 (2.73)	25 (2.65)	2 (0.62)	5 (13.16)	0 (0.00)	31 (2.77)	15 (13.04)
2.00	107 (3.75)	19 (2.01)	1 (0.31)	0 (0.00)	60 (22.22)	18 (1.61)	9 (7.83)
2.50	1005 (35.19)	357 (37.82)	193 (60.31)	3 (7.89)	7 (2.59)	425 (37.91)	19 (16.52)
3.00	168 (5.88)	61 (6.46)	6 (1.88)	1 (2.63)	5 (1.85)	70 (6.24)	25 (21.74)
3.75	14 (0.49)	6 (0.64)	2 (0.62)	0 (0.00)	0 (0.00)	6 (0.54)	0 (0.00)

DOI: 10.2174/0115734056472787260516112449, 2026, *22*, e15734056472787

Variables	Total (n = 2856)	Abdomen_CT_date_has_label (n = 944)	Abdomen_CT-1k_Fullysupervised_subtask1 (n = 321)	Abdomen_CT-1k_Semi (n = 38)	AMOS (n = 270)	Flare23 (n = 1121)	RSNA (n = 115)
4.00	34 (1.19)	14 (1.48)	2 (0.62)	4 (10.53)	0 (0.00)	14 (1.25)	0 (0.00)
5.00	700 (24.51)	209 (22.14)	36 (11.25)	22 (57.89)	193 (71.48)	236 (21.05)	4 (3.48)

Note: Values are reported as n (%). AP, arterial phase; PVP, portal venous phase; DP, delayed phase; NOC, non-contrast CT. Percentages are within each column. Slice thickness categories refer to reconstructed slice thickness. Missing/Unknown indicates unavailable metadata.

Table S2. Baseline characteristics and CT acquisition parameters of Dataset 2 (OutValidationSet; n=3,490 CT images) stratified by sex.

Variables	Total(n=3490)	Male(n=1939)	Female(n=1551)	P
Age, years	64.00 (53.00, 71.00)	65.00 (56.00, 72.00)	61.00 (51.00, 70.00)	<0.001
CT phase				0.311
NOC	862 (24.7%)	473 (24.4%)	389 (25.1%)	
AP	744 (21.3%)	427 (22.0%)	317 (20.4%)	
PVP	947 (27.1%)	506 (26.1%)	441 (28.4%)	
DP	937 (26.8%)	533 (27.5%)	404 (26.0%)	
Slice thickness (mm)				<0.001
0.6	6 (0.2%)	5 (0.3%)	1 (0.1%)	
0.625	3 (0.1%)	1 (0.1%)	2 (0.1%)	
1	253 (7.2%)	92 (4.7%)	161 (10.4%)	
1.25	3227 (92.5%)	1840 (94.9%)	1387 (89.4%)	
1.5	1 (0.0%)	1 (0.1%)	0 (0.0%)	
Manufacturer				<0.001
GE MEDICAL SYSTEMS	3230 (92.6%)	1841 (94.9%)	1389 (89.6%)	
NMS	135 (3.9%)	7 (0.4%)	128 (8.3%)	
SIEMENS	120 (3.4%)	90 (4.6%)	30 (1.9%)	
Philips	5 (0.1%)	1 (0.1%)	4 (0.3%)	
Tube voltage (kVp)	120.00 (120.00, 120.00)	120.00 (120.00, 120.00)	120.00 (120.00, 120.00)	0.013
Tube current (mA)	300.00 (300.00, 300.75)	300.00 (300.00, 326.00)	300.00 (300.00, 300.00)	<0.001

Footnote: Values are presented as n (%) for categorical variables and median (IQR) for continuous variables. CT images denote phase-specific CT image series; CT examinations are case-level. Abbreviations: NOC, non-contrast; AP, arterial phase; PVP, portal venous phase; DP, delayed phase.

Table S3. Source-specific segmentation performance on the aggregated public dataset and its labeled subsets.

Variables	Total (n=2856)	Abdomen_CT date_has_label (n=947)	Abdomen_CT-1k Fullysupervised subtask1 (n=322)	Abdomen_CT-1k Semi (n=74)	AMOS (n=270)	Flare23 (n=1127)	RSNA (n=116)
DSC, median (Q1, Q3)	0.98 (0.98, 0.99)	0.98 (0.98, 0.99)	0.98 (0.98, 0.99)	0.99 (0.98, 0.99)	0.97 (0.97, 0.98)	0.98 (0.98, 0.99)	0.98 (0.97, 0.98)
VS, median (Q1, Q3)	1.00 (0.99, 1.00)	1.00 (0.99, 1.00)	1.00 (0.99, 1.00)	0.99 (0.99, 1.00)	0.99 (0.99, 1.00)	1.00 (0.99, 1.00)	0.99 (0.99, 1.00)
HD (mm), median (Q1, Q3)	0.01 (0.01, 0.02)	0.01 (0.01, 0.02)	0.01 (0.01, 0.01)	0.01 (0.01, 0.01)	0.02 (0.01, 0.03)	0.01 (0.01, 0.02)	0.02 (0.01, 0.02)

Note: Metrics are reported as median (Q1, Q3). DSC, Dice similarity coefficient; VS, volume similarity; HD, Hausdorff distance.

Table S4. Post-hoc comparisons for segmentation performance across splits (raw and FDR-adjusted *p*-values).

Parameter	Comparison	Mann–Whitney U	Raw *p*	FDR-adjusted *p*	Interpretation
Dice similarity coefficient	External validation set vs Testing set	982,248	<0.001	<0.001	Significant after FDR
Dice similarity coefficient	External validation set vs Training set	7,924,302	<0.001	<0.001	Significant after FDR
Dice similarity coefficient	External validation set vs Validation set	968,409	<0.001	<0.001	Significant after FDR
Dice similarity coefficient	Testing set vs Training set	298,804	0.024	0.029	Significant after FDR
Dice similarity coefficient	Testing set vs Validation set	39,982	0.909	0.909	Not significant after FDR
Dice similarity coefficient	Training set vs Validation set	348,358	0.019	0.029	Significant after FDR
Volume similarity	External validation set vs Testing set	978,826	<0.001	<0.001	Significant after FDR
Volume similarity	External validation set vs Training set	7,895,796	<0.001	<0.001	Significant after FDR
Volume similarity	External validation set vs Validation set	964,726	<0.001	<0.001	Significant after FDR
Volume similarity	Testing set vs Training set	301,612	0.044	0.065	Not significant after FDR
Volume similarity	Testing set vs Validation set	37,650	0.276	0.331	Not significant after FDR
Volume similarity	Training set vs Validation set	326,253	0.647	0.647	Not significant after FDR
Hausdorff distance (mm)	External validation set vs Testing set	15,061	<0.001	<0.001	Significant after FDR
Hausdorff distance (mm)	External validation set vs Training set	124,630	<0.001	<0.001	Significant after FDR
Hausdorff distance (mm)	External validation set vs Validation set	15,020	<0.001	<0.001	Significant after FDR
Hausdorff distance (mm)	Testing set vs Training set	380,976	<0.001	<0.001	Significant after FDR
Hausdorff distance (mm)	Testing set vs Validation set	39,922	0.934	0.934	Not significant after FDR
Hausdorff distance (mm)	Training set vs Validation set	269,460	<0.001	<0.001	Significant after FDR
Average Hausdorff distance (mm)	External validation set vs Testing set	12,441	<0.001	<0.001	Significant after FDR
Average Hausdorff distance (mm)	External validation set vs Training set	102,631	<0.001	<0.001	Significant after FDR
Average Hausdorff distance (mm)	External validation set vs Validation set	12,269	<0.001	<0.001	Significant after FDR
Average Hausdorff distance (mm)	Testing set vs Training set	356,164	0.009	0.011	Significant after FDR
Average Hausdorff distance (mm)	Testing set vs Validation set	39,202	0.773	0.773	Not significant after FDR
Average Hausdorff distance (mm)	Training set vs Validation set	285,714	0.003	0.004	Significant after FDR

Footnotes: 1. Pairwise tests were adjusted within each metric using the Benjamini–Hochberg FDR procedure.
2. "Significant after FDR" should be used only when the adjusted *p*-value is < 0.05.

Table S5. Phase-stratified sex comparisons (raw and FDR-adjusted *p*-values).

Variable	All	-	-	-	-	NoC	-	-	-	-	AP	-	-	-	-	PVP	-	-	-	-	DP	-	-	-	-
	**Male**	**Female**	**Mann–Whitney U**	**Raw *p***	**FDR-adjusted *p***	**Male**	**Female**	**Mann–Whitney U**	**Raw *p***	**FDR-adjusted *p***	**Male**	**Female**	**Mann–Whitney U**	**Raw *p***	**FDR-adjusted *p***	**Male**	**Female**	**Mann–Whitney U**	**Raw *p***	**FDR-adjusted *p***	**Male**	**Female**	**Mann–Whitney U**	**Raw *p***	**FDR-adjusted *p***
Count	N=1939	N=1551	-	-	-	N=473	N=389	-	-	-	N=427	N=317	-	-	-	N=506	N=441	-	-	-	N=533	N=404	-	-	-
Average CT value (HU)	N=1939	N=1551	1,297,301	<0.001	<0.001	N=473	N=389	96,145	0.254	0.254	N=427	N=317	39,127	<0.001	<0.001	N=506	N=441	76,560	<0.001	<0.001	N=533	N=404	84,697	<0.001	<0.001
Spleen volume (cm^3)	210.42 [160.55, 276.41]	161.68 [125.30, 216.02]	2,018,213	<0.001	<0.001	206.32 [157.23, 274.71]	157.55 [122.56, 209.96]	123,943	<0.001	<0.001	204.18 [157.50, 270.18]	158.87 [124.47, 212.02]	91,044	<0.001	<0.001	213.74 [158.40, 284.88]	163.70 [125.99, 217.72]	149,412	<0.001	<0.001	215.14 [169.59, 278.78]	167.94 [127.98, 226.22]	143,889	<0.001	<0.001
x diameter (cm)	9.18 [8.40, 9.95]	8.54 [7.82, 9.22]	1,999,402	<0.001	<0.001	9.13 [8.44, 9.95]	8.44 [7.81, 9.17]	123,086	<0.001	<0.001	9.13 [8.18, 9.79]	8.59 [7.80, 9.23]	86,484	<0.001	<0.001	9.20 [8.40, 9.95]	8.41 [7.72, 9.13]	151,433	<0.001	<0.001	9.24 [8.48, 9.98]	8.65 [7.93, 9.28]	143,818	<0.001	<0.001
y diameter (cm)	9.37 [8.07, 10.73]	8.82 [7.58, 10.12]	1,777,732	<0.001	<0.001	9.25 [7.81, 10.62]	8.75 [7.56, 10.12]	106,388	<0.001	<0.001	9.34 [8.21, 10.67]	8.72 [7.51, 9.98]	82,694	<0.001	<0.001	9.48 [8.07, 10.83]	8.86 [7.63, 10.15]	130,652	<0.001	<0.001	9.45 [8.13, 10.74]	8.83 [7.59, 10.22]	127,925	<0.001	<0.001
z diameter (cm)	9.40 [8.05, 10.70]	8.50 [7.40, 9.70]	1,895,192	<0.001	<0.001	9.30 [8.00, 10.70]	8.40 [7.40, 9.30]	119,674	<0.001	<0.001	9.50 [7.99, 10.75]	8.50 [7.50, 9.50]	85,112	<0.001	<0.001	9.40 [8.10, 10.67]	8.60 [7.40, 9.80]	137,989	<0.001	<0.001	9.50 [8.10, 10.80]	8.57 [7.60, 9.80]	134,318	<0.001	<0.001

Notes: 1. All continuous variables are reported as median
[Q1, Q3]. Counts are shown as N.
2. Male vs female comparisons within each phase were performed using two-sided Mann–Whitney U tests.
3. FDR-adjusted *p*-values were calculated across all 25 tests in Table 2 (5 variables × 5 strata).
4. A result should be described as statistically significant only when the FDR-adjusted *p*-value is < 0.05.
5. The U statistic can be numerically large because it scales with sample size and tied ranks; it is not an effect-size measure.

Table S6a. Age-stratified sex comparisons (raw and FDR-adjusted *p*-values).

Variable	All	-	-	-	-	18-27	-	-	-	-	28-37	-	-	-	-	38-47	-	-	-	-
	**Male**	**Female**	**Mann–Whitney U**	**Raw *p***	**FDR-adjusted *p***	**Male**	**Female**	**Mann–Whitney U**	**Raw *p***	**FDR-adjusted *p***	**Male**	**Female**	**Mann–Whitney U**	**Raw *p***	**FDR-adjusted *p***	**Male**	**Female**	**Mann–Whitney U**	**Raw *p***	**FDR-adjusted *p***
Count	N=1939	N=1551	-	-	-	N=37	N=44	-	-	-	N=67	N=129	-	-	-	N=132	N=141	-	-	-
Average CT value (HU)	N=1939	N=1551	1,297,301	<0.001	<0.001	N=37	N=44	656	0.135	0.146	N=67	N=129	3,650	0.075	0.091	N=132	N=141	7,858	0.026	0.033
Spleen volume (cm^3)	210.42 [160.55, 276.41]	161.68 [125.30, 216.02]	2,018,213	<0.001	<0.001	252.21 [189.05, 283.78]	227.30 [180.55, 256.33]	976	0.126	0.140	274.20 [235.50, 400.62]	172.04 [147.32, 222.47]	7,335	<0.001	<0.001	232.22 [194.61, 308.98]	182.20 [140.88, 235.21]	13,201	<0.001	<0.001
x diameter (cm)	9.18 [8.40, 9.95]	8.54 [7.82, 9.22]	1,999,402	<0.001	<0.001	9.18 [8.23, 9.70]	8.91 [8.37, 9.71]	862	0.649	0.649	9.98 [9.27, 10.68]	8.36 [7.95, 9.02]	7,446	<0.001	<0.001	9.47 [8.83, 10.15]	8.35 [7.85, 9.29]	14,130	<0.001	<0.001
y diameter (cm)	9.37 [8.07, 10.73]	8.82 [7.58, 10.12]	1,777,732	<0.001	<0.001	10.36 [9.02, 11.48]	9.97 [9.22, 10.89]	873	0.579	0.594	10.90 [9.54, 12.11]	9.30 [8.44, 10.39]	6,431	<0.001	<0.001	9.77 [8.63, 11.02]	9.50 [8.25, 10.44]	10,404	0.092	0.108
z diameter (cm)	9.40 [8.05, 10.70]	8.50 [7.40, 9.70]	1,895,192	<0.001	<0.001	10.30 [8.30, 11.20]	9.75 [9.12, 10.30]	981	0.114	0.130	10.40 [9.40, 11.65]	9.00 [8.10, 9.70]	6,772	<0.001	<0.001	9.90 [8.80, 11.22]	8.70 [8.00, 9.70]	12,740	<0.001	<0.001

Variable	48-57	-	-	-	-	58-67	-	-	-	-	68-77	-	-	-	-	≥78	-	-	-	-
	**Male**	**Female**	**Mann–Whitney U**	**Raw *p***	**FDR-adjusted *p***	**Male**	**Female**	**Mann–Whitney U**	**Raw *p***	**FDR-adjusted *p***	**Male**	**Female**	**Mann–Whitney U**	**Raw *p***	**FDR-adjusted *p***	**Male**	**Female**	**Mann–Whitney U**	**Raw *p***	**FDR-adjusted *p***
Count	N=297	N=286	-	-	-	N=602	N=418	-	-	-	N=588	N=386	-	-	-	N=216	N=147	-	-	-
Average CT value (HU)	N=297	N=286	35,741	<0.001	0.001	N=602	N=418	103,965	<0.001	<0.001	N=588	N=386	102,538	0.011	0.014	N=216	N=147	14,703	0.232	0.244
Spleen volume (cm^3)	232.82 [187.61, 294.54]	190.22 [145.53, 252.30]	55,319	<0.001	<0.001	210.61 [161.36, 277.61]	158.40 [122.58, 210.18]	172,844	<0.001	<0.001	191.30 [144.09, 252.83]	144.46 [104.98, 178.64]	159,866	<0.001	<0.001	181.92 [136.68, 234.24]	132.92 [103.06, 179.01]	22,064	<0.001	<0.001
x diameter (cm)	9.18 [8.46, 10.22]	8.75 [8.12, 9.35]	53,111	<0.001	<0.001	9.14 [8.40, 9.96]	8.54 [7.82, 9.26]	165,052	<0.001	<0.001	9.08 [8.25, 9.80]	8.37 [7.55, 9.03]	153,578	<0.001	<0.001	9.10 [8.22, 9.67]	8.31 [7.66, 9.15]	20,929	<0.001	<0.001
y diameter (cm)	9.98 [8.93, 10.88]	9.14 [8.33, 10.70]	49,120	0.001	0.001	9.30 [7.97, 10.89]	8.63 [7.36, 10.03]	152,848	<0.001	<0.001	9.13 [7.83, 10.39]	8.07 [7.04, 9.26]	145,974	<0.001	<0.001	8.89 [7.53, 9.80]	8.37 [7.00, 9.35]	18,557	0.006	0.008
z diameter (cm)	10.10 [8.60, 11.10]	9.10 [8.00, 10.67]	52,346	<0.001	<0.001	9.50 [8.30, 10.80]	8.50 [7.50, 9.40]	169,174	<0.001	<0.001	9.00 [7.70, 10.30]	7.85 [7.00, 9.30]	145,000	<0.001	<0.001	8.30 [7.30, 10.03]	7.80 [6.50, 8.76]	19,884	<0.001	<0.001

Notes: 1. All continuous variables are reported as median
[Q1, Q3]. Counts are shown as N.
2. Male vs female comparisons within each age group were performed using two-sided Mann–Whitney U tests.
3. FDR-adjusted *p*-values were calculated across all 40 tests in Table 3 (5 variables × 8 strata).
4. Use the adjusted *p*-value for manuscript wording; if raw $p < 0.05$ but adjusted $p \geq 0.05$, state “not significant after FDR correction”.
5. Units: HU for CT attenuation, cm^3 for spleen volume, and cm for x/y/z diameters.

Table S6B. Additional age strata and calculation notes.

Family	Parameter	Stratum	Male Summary	Female Summary	Raw *p*	FDR-adjusted *p*	Use this Wording
Phase-by-sex	Average CT value (HU)	All	-	-	<0.001	<0.001	Significant after FDR
Phase-by-sex	Average CT value (HU)	NoC	-	-	0.254	0.254	Not significant after FDR
Phase-by-sex	Average CT value (HU)	AP	-	-	<0.001	<0.001	Significant after FDR
Phase-by-sex	Average CT value (HU)	PVP	-	-	<0.001	<0.001	Significant after FDR
Phase-by-sex	Average CT value (HU)	DP	-	-	<0.001	<0.001	Significant after FDR
Phase-by-sex	Spleen volume (cm^3)	All	210.42 [160.55, 276.41]	161.68 [125.30, 216.02]	<0.001	<0.001	Significant after FDR
Phase-by-sex	Spleen volume (cm^3)	NoC	206.32 [157.23, 274.71]	157.55 [122.56, 209.96]	<0.001	<0.001	Significant after FDR
Phase-by-sex	Spleen volume (cm^3)	AP	204.18 [157.50, 270.18]	158.87 [124.47, 212.02]	<0.001	<0.001	Significant after FDR
Phase-by-sex	Spleen volume (cm^3)	PVP	213.74 [158.40, 284.88]	163.70 [125.99, 217.72]	<0.001	<0.001	Significant after FDR
Phase-by-sex	Spleen volume (cm^3)	DP	215.14 [169.59, 278.78]	167.94 [127.98, 226.22]	<0.001	<0.001	Significant after FDR
Phase-by-sex	x diameter (cm)	All	9.18 [8.40, 9.95]	8.54 [7.82, 9.22]	<0.001	<0.001	Significant after FDR
Phase-by-sex	x diameter (cm)	NoC	9.13 [8.44, 9.95]	8.44 [7.81, 9.17]	<0.001	<0.001	Significant after FDR
Phase-by-sex	x diameter (cm)	AP	9.13 [8.18, 9.79]	8.59 [7.80, 9.23]	<0.001	<0.001	Significant after FDR
Phase-by-sex	x diameter (cm)	PVP	9.20 [8.40, 9.95]	8.41 [7.72, 9.13]	<0.001	<0.001	Significant after FDR
Phase-by-sex	x diameter (cm)	DP	9.24 [8.48, 9.98]	8.65 [7.93, 9.28]	<0.001	<0.001	Significant after FDR
Phase-by-sex	y diameter (cm)	All	9.37 [8.07, 10.73]	8.82 [7.58, 10.12]	<0.001	<0.001	Significant after FDR
Phase-by-sex	y diameter (cm)	NoC	9.25 [7.81, 10.62]	8.75 [7.56, 10.12]	<0.001	<0.001	Significant after FDR
Phase-by-sex	y diameter (cm)	AP	9.34 [8.21, 10.67]	8.72 [7.51, 9.98]	<0.001	<0.001	Significant after FDR
Phase-by-sex	y diameter (cm)	PVP	9.48 [8.07, 10.83]	8.86 [7.63, 10.15]	<0.001	<0.001	Significant after FDR
Phase-by-sex	y diameter (cm)	DP	9.45 [8.13, 10.74]	8.83 [7.59, 10.22]	<0.001	<0.001	Significant after FDR
Phase-by-sex	z diameter (cm)	All	9.40 [8.05, 10.70]	8.50 [7.40, 9.70]	<0.001	<0.001	Significant after FDR
Phase-by-sex	z diameter (cm)	NoC	9.30 [8.00, 10.70]	8.40 [7.40, 9.30]	<0.001	<0.001	Significant after FDR
Phase-by-sex	z diameter (cm)	AP	9.50 [7.99, 10.75]	8.50 [7.50, 9.50]	<0.001	<0.001	Significant after FDR
Phase-by-sex	z diameter (cm)	PVP	9.40 [8.10, 10.67]	8.60 [7.40, 9.80]	<0.001	<0.001	Significant after FDR
Phase-by-sex	z diameter (cm)	DP	9.50 [8.10, 10.80]	8.57 [7.60, 9.80]	<0.001	<0.001	Significant after FDR
Age-by-sex	Average CT value (HU)	All	-	-	<0.001	<0.001	Significant after FDR
Age-by-sex	Average CT value (HU)	18-27	-	-	0.135	0.146	Not significant after FDR
Age-by-sex	Average CT value (HU)	28-37	-	-	0.075	0.091	Not significant after FDR
Age-by-sex	Average CT value (HU)	38-47	-	-	0.026	0.033	Significant after FDR
Age-by-sex	Average CT value (HU)	48-57	-	-	<0.001	0.001	Significant after FDR
Age-by-sex	Average CT value (HU)	58-67	-	-	<0.001	<0.001	Significant after FDR
Age-by-sex	Average CT value (HU)	68-77	-	-	0.011	0.014	Significant after FDR
Age-by-sex	Average CT value (HU)	≥78	-	-	0.232	0.244	Not significant after FDR
Age-by-sex	Spleen volume (cm^3)	All	210.42 [160.55, 276.41]	161.68 [125.30, 216.02]	<0.001	<0.001	Significant after FDR

Family	Parameter	Stratum	Male Summary	Female Summary	Raw *p*	FDR-adjusted *p*	Use this Wording
Age-by-sex	Spleen volume (cm^3)	18-27	252.21 [189.05, 283.78]	227.30 [180.55, 256.33]	0.126	0.140	Not significant after FDR
Age-by-sex	Spleen volume (cm^3)	28-37	274.20 [235.50, 400.62]	172.04 [147.32, 222.47]	<0.001	<0.001	Significant after FDR
Age-by-sex	Spleen volume (cm^3)	38-47	232.22 [194.61, 308.98]	182.20 [140.88, 235.21]	<0.001	<0.001	Significant after FDR
Age-by-sex	Spleen volume (cm^3)	48-57	232.82 [187.61, 294.54]	190.22 [145.53, 252.30]	<0.001	<0.001	Significant after FDR
Age-by-sex	Spleen volume (cm^3)	58-67	210.61 [161.36, 277.61]	158.40 [122.58, 210.18]	<0.001	<0.001	Significant after FDR
Age-by-sex	Spleen volume (cm^3)	68-77	191.30 [144.09, 252.83]	144.46 [104.98, 178.64]	<0.001	<0.001	Significant after FDR
Age-by-sex	Spleen volume (cm^3)	≥78	181.92 [136.68, 234.24]	132.92 [103.06, 179.01]	<0.001	<0.001	Significant after FDR
Age-by-sex	x diameter (cm)	All	9.18 [8.40, 9.95]	8.54 [7.82, 9.22]	<0.001	<0.001	Significant after FDR
Age-by-sex	x diameter (cm)	18-27	9.18 [8.23, 9.70]	8.91 [8.37, 9.71]	0.649	0.649	Not significant after FDR
Age-by-sex	x diameter (cm)	28-37	9.98 [9.27, 10.68]	8.36 [7.95, 9.02]	<0.001	<0.001	Significant after FDR
Age-by-sex	x diameter (cm)	38-47	9.47 [8.83, 10.15]	8.35 [7.85, 9.29]	<0.001	<0.001	Significant after FDR
Age-by-sex	x diameter (cm)	48-57	9.18 [8.46, 10.22]	8.75 [8.12, 9.35]	<0.001	<0.001	Significant after FDR
Age-by-sex	x diameter (cm)	58-67	9.14 [8.40, 9.96]	8.54 [7.82, 9.26]	<0.001	<0.001	Significant after FDR
Age-by-sex	x diameter (cm)	68-77	9.08 [8.25, 9.80]	8.37 [7.55, 9.03]	<0.001	<0.001	Significant after FDR
Age-by-sex	x diameter (cm)	≥78	9.10 [8.22, 9.67]	8.31 [7.66, 9.15]	<0.001	<0.001	Significant after FDR
Age-by-sex	y diameter (cm)	All	9.37 [8.07, 10.73]	8.82 [7.58, 10.12]	<0.001	<0.001	Significant after FDR
Age-by-sex	y diameter (cm)	18-27	10.36 [9.02, 11.48]	9.97 [9.22, 10.89]	0.579	0.594	Not significant after FDR
Age-by-sex	y diameter (cm)	28-37	10.90 [9.54, 12.11]	9.30 [8.44, 10.39]	<0.001	<0.001	Significant after FDR
Age-by-sex	y diameter (cm)	38-47	9.77 [8.63, 11.02]	9.50 [8.25, 10.44]	0.092	0.108	Not significant after FDR
Age-by-sex	y diameter (cm)	48-57	9.98 [8.93, 10.88]	9.14 [8.33, 10.70]	0.001	0.001	Significant after FDR
Age-by-sex	y diameter (cm)	58-67	9.30 [7.97, 10.89]	8.63 [7.36, 10.03]	<0.001	<0.001	Significant after FDR
Age-by-sex	y diameter (cm)	68-77	9.13 [7.83, 10.39]	8.07 [7.04, 9.26]	<0.001	<0.001	Significant after FDR
Age-by-sex	y diameter (cm)	≥78	8.89 [7.53, 9.80]	8.37 [7.00, 9.35]	0.006	0.008	Significant after FDR
Age-by-sex	z diameter (cm)	All	9.40 [8.05, 10.70]	8.50 [7.40, 9.70]	<0.001	<0.001	Significant after FDR
Age-by-sex	z diameter (cm)	18-27	10.30 [8.30, 11.20]	9.75 [9.12, 10.30]	0.114	0.130	Not significant after FDR
Age-by-sex	z diameter (cm)	28-37	10.40 [9.40, 11.65]	9.00 [8.10, 9.70]	<0.001	<0.001	Significant after FDR
Age-by-sex	z diameter (cm)	38-47	9.90 [8.80, 11.22]	8.70 [8.00, 9.70]	<0.001	<0.001	Significant after FDR
Age-by-sex	z diameter (cm)	48-57	10.10 [8.60, 11.10]	9.10 [8.00, 10.67]	<0.001	<0.001	Significant after FDR
Age-by-sex	z diameter (cm)	58-67	9.50 [8.30, 10.80]	8.50 [7.50, 9.40]	<0.001	<0.001	Significant after FDR
Age-by-sex	z diameter (cm)	68-77	9.00 [7.70, 10.30]	7.85 [7.00, 9.30]	<0.001	<0.001	Significant after FDR

Family	Parameter	Stratum	Male Summary	Female Summary	Raw *p*	FDR-adjusted *p*	Use this Wording
Age-by-sex	z diameter (cm)	≥78	8.30 [7.30, 10.03]	7.80 [6.50, 8.76]	<0.001	<0.001	Significant after FDR

Notes: 1. This helper sheet is intended to prevent discrepancies between narrative text and reported *p*-values.
2. Only rows with FDR-adjusted $p < 0.05$ should be described as statistically significant.
Test statistics are Mann–Whitney U (Wilcoxon rank-sum) for two-group comparisons. *p*-values are two-sided. FDR-adjusted *p*-values were computed using the Benjamini–Hochberg procedure within each block.

© 2026 The Author(s). Published by Bentham Science Publisher.

This is an open access article distributed under the terms of the Creative Commons Attribution 4.0 International Public License (CC-BY 4.0), a copy of which is available at: https://creativecommons.org/licenses/by/4.0/legalcode. This license permits unrestricted use, distribution, and reproduction in any medium, provided the original author and source are credited.

HOW TO CITE:
Tan T, Zhang Y, Zhang X, Wang X. Deep-Learning-based Automatic Segmentation and Quantitative Measurement of Normal Spleen in Chinese Adults. Curr Med Imaging, 2026; 22: e15734056472787.
http://dx.doi.org/10.2174/0115734056472787260516112449
